# First‐line nurse managers’ perceptions of the challenges involved in decision‐making when older patients’ wish to die

**DOI:** 10.1002/nop2.131

**Published:** 2018-02-28

**Authors:** Anne Lise Holm, Astrid Karin Berland, Arvid Karl Birkeland, Elisabeth Severinsson

**Affiliations:** ^1^ Faculty of Health and Social Sciences Western Norway University of Applied Sciences Haugesund Norway; ^2^ Faculty of Health and Social Sciences Centre for Women's, Family and Child Health University College Southeast Norway Kongsberg Norway

**Keywords:** death wishes, decision‐making, first‐line nurse manager, older patients

## Abstract

**Aim:**

The aim of this study was to explore first‐line nurse managers’ perceptions of the challenges involved in decision‐making concerning older patients who wish to die.

**Design:**

A descriptive qualitative design in three communities in Norway between January 2016 and June 2016.

**Methods:**

Qualitative focus group interviews with eight first‐line nurse managers.

**Results:**

One main theme and two themes were identified: Struggling to make the right decision, The challenge of social isolation and loneliness and When life becomes too painful and problematic. The sub‐themes revealed that: Arranging social meeting places where the older patients can talk to other older people is crucial. The participants explained that it was not an easy task to gain an overview of the older patients situation. The right decision could be encouraging the patients to talk about their problems by giving them more time, thereby showing that somebody cares about them.

## 1 INTRODUCTION

Research indicates that death wishes often occur among older people (Ayalon & Shiovitz‐Ezra, 2011; van Winjgaarden, Leget, & Goossensen 2014). In Europe, 10%–20% of older people have or have had thoughts about death and no longer wish to live (Rurup, Deeg, Poppellaars, Kerkhof, & Onwuteaka‐Philipsen 2011). Epidemiological and prevalence studies have found a 10%–20% association between death wishes and mental disorders (Rurup, Deeg et al., 2011; van Winjgaarden et al. 2014). Rurup, Deeg et al. (2011) revealed that 20% of people with a wish to die suffered from a depressive disorder. However, 5%–30% of older people who wish to die have no psychiatric or depressive disorder (Rurup, Deeg et al., 2011; van Wijngaarden, Leget, & Goossensen, 2015). Most older people who wish to die probably consider suicide unacceptable, as it is associated with despair and mutilation (van Wijngaarden, Leget, & Goossensen 2016). However, differences between older patients who wish to die and those who are suicidal have been described (Kim, Bogner, Brown, & Gallo, 2006).

The basis of home health care in Norway is the provision of healthcare to individuals of all ages and first‐line nurse managers (FLNMs) are responsible for overseeing first level nursing services. In the literature the term FLNM has been used interchangeably with “head nurse”, “ward sister”, “ward manager, “nursing manager” and “ward leader” (Finkelman, 2006; Gunawan & Aungsuroch, 2017; Wynne, 2003). However, there seems to be a tendency for Registered Nurses to withdraw from their basic nursing duties when they become FLNMs (Johansson, Andersson, Gustafsson, & Sandahl 2010). Three studies on elder care in Sweden described FLNMs’ experiences of elder care funding (Antonsson, Eriksson Korjonen, & Rosengren, 2012), male FLNMs experiences of the work situation (Hagerman, Engström, Häggström, Wadensten, & Skytt, 2015) and FLNMs**’** healthcare professional's descriptions of different structures in health care (Skytt, Hagerman, Strömberg, & Engström, 2015). According to Gunawan and Aungsuroch (2017), the attributes of FLNMs’ managerial competence are self‐development, planning, organizing, leading, managing legal issues, managing ethical issues and delivering health care. First‐line nurse managers have a great impact on improving the quality of care and patient outcomes (Gunawan & Aungsuroch, 2017). They are responsible for translating strategic goals, providing administrative and clinical leadership, as well as having 24‐hour accountability for all patient care activities on the unit (Thrall, 2006).

### 1.1 Background

In line with Johansson, Pörn, and Gustafsson (2007), we found that FLNMs often lack initiative, competence and ability for decision‐making concerning older patients who wish to die, when they perceived themselves as a leader or manager. Death statements are often termed “death talk” or “suicide talk” and described in the literature as “desire to die statements” (Breitbart, Chocinov, & Passik, 2004; Hudson et al., 2006). Such ‘desire to die statements can be confusing for FLNMs in the decision‐making of older patients. According to Hudson, Schofield, Kelly, Hudson, Street, O’Connor, Kristinjanson, Ashby, & Aranda, (2006), one should respond to ‘desire of death statements’ with compassion and professionalism, regardless of these statements might be rational or reasonable in some circumstances. The existential impact of age related loss plays an important role in the development of a wish to die (van Wijngaarden et al., 2016; van Winjgaarden et al., 2014). Other contributory factors are: personal characteristics including low perceived mastery, involuntary urine loss, having a speech impairment, financial problems, loneliness, a small social network, being divorced, as well as certain perceptions and values (Rurup, Deeg et al., 2011; van Winjgaarden et al., 2014).

Research in elder care has few studies described FLNMs specific problems in decision‐making of groups of elderly patients as older patients with death wishes. The aim of the study was; to explore first‐line nurse managers' perceptions of the challenges involved in decision‐making concerning older patients who wish to die.

## 2 THE STUDY

### 2.1 Design

This study used a descriptive qualitative design.

### 2.2 Sample and setting

The sample consisted of eight female FLNMs (*n *=* *8) to obtain a range of perceptions (Polit & Beck, 2012). The FLNMs worked in the healthcare services in three communities. Additional demographic characteristics are presented in Table 1.

**Table 1:** Demographic characteristics of the first‐line nurse managers perceptions

Community (*n *=* *3)	Age (*n *=* *8)	Sex	Length of time as first‐line nurse manager	Education
A	41	Female	2 years	Gastro nursing, palliative care
A	46	Female	4 years	Mental health nursing, social pedagogics
A	26	Female	6 months	Registered Nurse
B	46	Female	6 months	Geriatric nursing, administrative education
B	51	Female	6 months	Cancer nursing, administrative education
C	55	Female	2 years	Geriatric nursing, administrative education
C	60	Female	Over 10 years	Registered Nurse
C	54	Female	Over 10 years	Mental health and geriatric nursing, administrative education

In the recruitment process, leaders of 12 different communities were informed of the project in the course of a meeting arranged by a University College. The first author (ALH) described some of the challenges faced by FLNMs in caring for older patients who wish to die. The 12 communities were contacted by e‐mail in December, 2015. Four communities answered in a positive way but wanted more information about the project, which was sent in February, 2016. Unfortunately, three of the communities declined, thus a new request was sent to two of the other communities on the first e‐mail list. Finally, three communities agreed to participate, thus the focus group comprised eight participants: three first‐line nurse managers from community A, two from community B and three from community C.

### 2.3 Data collection

The study was based on qualitative focus group interviews (Morgan, 1997). Focus group interviews are characterized by carefully planned discussions on defined areas or topics. Group dynamics facilitate the exploration and clarification of views that might be less accessible or evident in individual interviews (Orvik, Larun, Berland, & Ringsberg, 2013). One focus group interview was performed, which lasted for almost 2 hr. The first author led the discussion and ensured that the dialogue between the participants was relevant to the theme. The role as co‐moderator (AKB, second author) was to make sure that the participants were allowed to speak for an equal length of time, observe the discussions and ascertain that the recording equipment was operating properly. The moderator (ALH) and co‐moderator (AKB) later discussed the different aspects that emerged in the observation and the discussion. The aim was to stimulate a discussion that was not influenced by the authors. The focus group interview method allows the participants to discuss different aspects of a theme as freely as possible (Morgan, 1997). The participants were asked to describe challenges in the decision‐making in their role as a FLNM. According to Morgan (1997), the moderator's role is to start the discussion in an open and general way. The moderator encouraged the participants to provide a rich description of their role in home health care in general, followed by questions about their care of and responsibility for older patients who had expressed the wish to die and were tired of living. Some of the participants were silent and the moderator tried to encourage them to contribute to the discussion. While the FLNMs in each of the communities knew each other, most had little knowledge of each other's working conditions. The content of the focus group interview was digitally recorded and transcribed verbatim by the first author.

### 2.4 Data analysis

This study used a thematic analysis (Polit & Beck, 2012), which is defined as ‘a method for identifying, analysing and reporting patterns (themes) in the data (Braun & Clarke, 2006 p. 79). The analysis of the transcripts was conducted by the first author (ALH). The first step involved exploring the text by reading it several times to find patterns (preliminary themes). The meaning units and preliminary themes were coded by the authors (ALH, AKB, AB, ES), who then discussed and revised the text to ensure agreement on main theme, themes and sub‐themes, after which the text was structured in the form of a table (Table 2).

**Table 2:** First‐line nurse managers’ perceptions of the challenges involved in decision‐making when older patients’ wish to die

Main theme	Struggling to make the right decision	
Themes	Sub‐themes	Statements
The challenge of social isolation and loneliness	Arranging social meeting places	“Arranging social meeting places can prevent anxiety and depression among the elderly”
When life becomes too painful and problematic	Gaining an overview of the situation	“Gaining an overview of the older patient's situation”
	Asking direct questions about death ideation	“My experience as an FLNM is that most older patients want to live. Asking direct questions about death wishes can constitute a turning point”

During the analysis process, the authors validated the text by discussing how it could be understood and interpreted (Table 2), after which they agreed on the selection of quotations to illustrate the data, as well as the rationale for the chosen order of presentation of the quotations.

### 2.5 Trustworthiness

The authors are responsible for ensuring the trustworthiness of the research process and the truthfulness of the thematic analysis. According to Lincoln and Cuba (1985), the trustworthiness of qualitative data is established by; credibility, transferability, dependability and confirmability. Credibility refers to confidence in the truth of the data, in addition to the reflection of multiple realities. Transferability concerns the appropriateness of the data in settings other than that of the study. In this process the authors used statements from the text to represent the participants’ perspectives. Confirmability refers to the objectivity or neutrality of the data (Polit & Beck, 2012), which means that the researcher must analyse the text as objectively as possible. Dependability is related to data stability over time and conditions (Polit & Beck, 2012).

### 2.6 Ethical consideration

The participants were informed about the purpose, method and their right to withdraw from the study. They were assured that the data would be treated confidentially and that their names would be removed from the transcripts (World Medical Association, 2013). The study was approved by the Norwegian Social Science Data Services (NSD) (No. 44782) and carried out in accordance with the Ethical guidelines for nursing research in the Nordic countries (Northern Nurses Federation, 2003).

## 3 RESULTS

One main theme: Struggling to make the right decision and two themes: The challenge of social isolation and loneliness and when life becomes too painful and problematic emerged.

### 3.1 Struggling to make the right decision

The participants struggling to make the right decision asking themselves if the older patients really meant that they wanted to die or if it could be an expression of physical or social needs and/or emotional or physical pain and distress. They had the impression that the wish to die was due to social isolation and loneliness, loss of husband/wife, friends, siblings and relatives. They often had to ask directly about whether the older patients really wanted to die. They often had to contact the general practitioner for a second opinion on the older patients’ situation.

### 3.2 The challenge of social isolation and loneliness

This theme could have emerged because the participants from community B revealed that they had just received a message from their leader that social isolation and loneliness constituted the new challenge in the community. The participants stated that the community wanted to organise more social meeting places for older people. The participant revealed that many of the older people in the community need to be included in a social milieu, even if they themselves do not seem to understand that they need help. One of the participants from community A stated that older patients had the opportunity to receive help from a social support worker once or twice a week. This social support worker assists people who are unable to function socially in everyday life with things such as shopping, going to the cinema or to a café:

“I think this is the social support that older patients need to survive. They need someone to confirm and see them”.

The participant reported that such an arrangement could be a question of life and death for an older patient who does not wish to live.

### 3.3 Arranging social meeting places

The participant from community C highlighted the fact that there has been too much focus on physical complaints and not enough on social factors related to older patients who do not want to live. Arranging social meeting‐places where they can talk to other older people is crucial. There often seemed to be too much focus on physical factors such as nutrition and bodily needs, while the social factors were ignored.

The participant from community B expressed the hope that a newly recruited coordinator could be contacted in relation to lonely older women, as problems arise when older people are left alone. The community wanted to offer organised social activities in the future. The participant hoped that the community would be able to solve this problem within a short time and stated:

“Arranging meeting places can prevent anxiety and depression among older patients”

### 3.4 When life becomes too painful and problematic

Two of the participants from communities, A and C have special responsibility for mental health. In their opinion, many factors such as loneliness, physical and/or emotional pain could be important when older patients express that they no longer wish to live. In other words, the desire for death could be related to the fact that life has become too painful and problematic.

### 3.5 Gaining an overview of the situation

The participants explained that when the older patients expressing a wish to die it was not an easy task to gain an overview of the situation. The participants rarely heard older patients directly stating that they wanted to take their own life, even in cases where they suffered a great deal of pain and were very ill. One reported that the older persons bear their burden with dignity, although they sometimes conceal their physical and emotional pain and problems. Another added:

“An old man states every day that he does not want to live anymore. However, he has just lost his wife so I suppose that he is in a grieving process. We have tried to help him, but when one is mourning it takes time”.

One participant from community B revealed that some older patients go through a crisis when diagnosed with a chronic disease. They become increasingly depressed and do not want to live anymore. The participant related that she asked them the following question; What can we do for you to make your life better? One of the participants from community B explained that in her position as a FLNM, it was not part of her role to visit the older patients in their own home, but sometimes she does. She stated that in one particular case:

“He took my arm and told me that he has much emotional pain and suffering. I asked if he had someone to talk to about this matter. He replied that the nurses who help him with showering are in a hurry and he could not bother them with his problems”

As FLNMs, they perceived death wishes as a cry for help and it was obvious that the older patients needed more help than they received. They sometimes had to refer older patients to the mental health service in the community.

### 3.6 Asking direct questions about death ideation

One participant from community A reported that older patients often show different signs of suffering. First‐line nurse managers must explore how older patients’ healthcare needs are met, as that can constitute a turning point and improve the situation. For example, one smoked in his apartment. The participant stated:

“We have to ask him directly if he has a plan for taking his own life. Sometimes he admits that he has”.

In 2012, community A established a team for mentally ill and older patients with an abuse problem, which has been of great help to the home healthcare staff. One participant explained that the team members try to help in times of crisis, adding that most crises can be avoided if the right decision is made. The right decision could be FLNMs encouraging older patients to talk about their problems by giving them more time, thereby showing that somebody cares about them. When offered a safer place, for example, in a special home for the mentally ill, their situation often improved. The participant stated that she often hears expressions such as: “I do not have enough strength to live any more. I need help”. Such statements are interpreted by the participants as something that has to be taken seriously:

“Most of these patients want to live. I believe that when you start talking about things that matter to them it can be a turning point”.

## 4 DISCUSSION

To explore first‐line nurse managers’ perceptions of the challenges involved in decision‐making concerning older patients who wish to die. The response of the FLNMs was interpreted as struggling to make the right decision. In recent years the healthcare organisations in Norway where the FLNMs are employed have been given more formal responsibility for staff. However, as communities in Norway are very independent and primary care is regulated by internal rules based on their budgets, some do not adhere to the new rules and have no staff member to perform the leadership function. Accordingly, FLNMs in Norway are responsible for carrying out the decisions made by the community healthcare administration office. A decision is based on information from significant others and/or healthcare professionals in the community.

As revealed by Johansson et al. (2007), FLNMs struggle to cope with their role as a nurse, an administrator and a leader. When describing their decision‐making they often appear to give precedence to their duties as a clinician or nurse. However, when responding to older patients’ death wishes, the FLNMs struggled to make the right decision, especially if they perceived themselves as a leader or manager, which is in accordance with Johansson et al. (2007). The FLNMs appeared to mainly assume the role of nurse clinician and lacked the perception that as a manager their duty is to bring problems to the attention of their leaders in the organisation. However, a later study by Johansson, Sandahl, and Hasson (2013) revealed that FLNMs were able to cope with highly demanding job situations because they had a relatively large degree of control over their work. The present findings do not support those of Johansson et al. (2013), as in our study the FLNMs did not seem to have much control over their role and work situation as managers and leaders.

The findings in this study revealed that the social determinants of death ideation are social isolation and loneliness. Most studies on the elderly and their wish to die have focused on individual and psychopathological models as opposed to social and community factors, while available research lacks reference to the environment and/or context (Saias, Beck, Bodard, Guignard, & du Roscoät, 2012). First‐line nurse managers’ need the competence to identify passive suicide in the elderly, often described as stopping medication prescribed for a chronic disease. Research on death ideation has increased over the last 20 years and seems to be related to risk factors such as poor health, sensory impairment (Saias et al., 2012), being widowed or single and low perceived social support. Loneliness is described as a discrepancy between expected support and the actual support received (Rurup et al., 2011; Saias et al., 2012). First‐line nurse managers need more knowledge about how to respond to older patients who exhibit depressive symptoms without having a diagnosis of depression. A study revealed that loneliness is of a subjective nature (Shiovitz‐Ezra & Leitsch, 2010). Homecare nurses often assume that the patients feel lonely, but the patients themselves did not feel lonely (Birkeland, 2013). Limited attention has been paid to the relationship between loneliness and death wishes in different age groups (Ayalon & Shiovitz‐Ezra, 2011).

The FLNMs described their role as a struggle due to having to make decisions both for themselves as well as on behalf of the healthcare professionals who make up the team in the community. They seemed to believe that the older patients’ life becomes too painful and problematic and asked themselves; what knowledge they need to alleviate the older patients’ emotional pain and suffering? It seems essential to explore the older patients’ emotional state and allocate sufficient time to enable the healthcare professional and older patient to reflect on these issues. This reflection will enable FLNMs and healthcare professionals to determine whether the wish to die should be taken seriously and can be related to emotional pain and suffering (Holm & Severinsson, 2011). The ability to talk about and reflect on experiences that constitute a suffering state can be important in a process that enables the mind to cope with the experiences. Asking direct questions about death ideation can be of the utmost importance when FLNMs have to make a decision based on information provided by healthcare professionals. Such direct questions can be related to death ideation or suicide plans, especially if the older patients have made death wishes. FLNMs must make a decision based on different situations that can occur in practice. Even if they gain a picture of the older patient's situation they may still find it difficult to make the right decision. They need to develop a working environment focused on the importance of listening to the inner experiences of older patients.

Older patients may believe that it is inappropriate to trouble FLNMs and healthcare professionals with their worries, or that healthcare professionals would be unable to help them as their feelings are unreasonable. Many factors may play a role in patients’ reluctance to disclose their death wishes because their life no longer has any meaning (Hudson et al., 2006). Death ideation can be viewed as reasonable reactions to unfavourable circumstances (Hudson et al., 2006).

### 4.1 Limitations

A limitation of the study is the authors’ reflection on the meaning of the statements in the light of their own experiences as mental health nurses (ALH, AB, ES), an anaesthesia nurse (AKB) and as researchers. It can be assumed that their pre‐understanding influenced the interpretation of the themes and sub‐themes (Gadamer, 2004). Ideally, the authors should have invited the participants to take part in a second focus group interview as recommended by Liamputtong (2011), but because of several work‐related problems the research group decided against asking them to participate in an additional focus group discussion.

### 4.2 Conclusion and implications for nursing management

The role of FLNMs has changed a great deal and future research should focus on the different challenges they encounter in decision‐making. The participants emphasized their everyday work as well as responsibility for their staff (Skytt, Ljunggren, Sjödén, & Carlsson, 2008). They need to provide a positive environment for patients which mean that they need greater authority, a wider mandate and to be more present on the ward (Skytt et al., 2008). Their professional frame of reference must be clearer. In addition, they require more competence and ability in their role of supervising staff and in taking the initiative to address the acute problems related to death wishes. They need to reflect on their leadership style and on how they can be empowered as administrators and leaders. Long‐term perspectives are necessary, leading to more strategic planning for care development (Johansson et al., 2010). The importance of FLNMs’ mentor and team building role has been confirmed (Zydziunaite & Suominen, 2014). Decision‐making and leadership styles can often be related to situational considerations. First‐line nurse managers could be effective leaders using their strengths and counteracting their weaknesses (Abualrub & Algamdi, 2012; Zydziunaite & Suominen, 2014), which would increase their decision‐making capacity and enable them to respond in elder care. Peer groups and supervisors are required to help them develop their ability as leaders and managers (Zydziunaite & Suominen, 2014).

However, FLNMs in Norway seem to find it difficult to decide whether to prioritise their role of clinician, manager or leader and do not appear to know what to do or who to turn to when they experience challenges in decision‐making in the organization (Lageson, 2004). They appear to speak both for themselves and on behalf of other healthcare professionals. Lack of initiative and competence can be a catastrophe, making them unable to respond as managers and leaders. They also need guidelines related to quality care and patient safety in community settings. Quality management principles should be familiar to the nursing staff (Lageson, 2004). Successful quality management is characterised by the involvement and empowerment of employees and mediated by their satisfaction with communication.

## AUTHOR CONTRIBUTIONS

ALH, Study design; ALH, AKB, data collection; ALH, AKB, AB, ES, data analysis, discussion and preparation.
